# GENDER DIFFERENCES ACROSS THE AGING SEMANTIC DIFFERENTIAL AND THE FRABONI SCALE OF AGEISM

**DOI:** 10.1093/geroni/igz038.316

**Published:** 2019-11-08

**Authors:** Robert C Intrieri, Rebecca A Dunterman

**Affiliations:** Western Illinois University, Macomb, Illinois, United States

## Abstract

The purpose of this study was to compare attitudes about aging between groups of raters categorized by gender, using the Aging Semantic Differential (ASD; Rosencrantz & McNevin, 1969) and the Fraboni Scale of Ageism (FSA, Fraboni, Saltstone, & Hughes, 1990). The current study assesses the relationships between the four factors of Aging Semantic Differential (ASD; Instrumentality, Autonomy, Acceptability, and Integrity) as well as the three factors of the Fraboni Scale of Ageism (FSA; Antilocution, Discrimination, and Avoidance) across gender groups. The convenience sample consisted of 471 undergraduate students, with a mean age of 19.68 (SD = 2.28). The mean age for men was 19.88 (SD = 3.31) and for women was 19.56 (SD = 1.37). A series of four ANOVAs were conducted using the ASD factors. Results showed significance for the ASD-Instrumental (F(1, 470) = 4.922, p = .027); ASD-Acceptability F(1, 470) = 8.616, p = .003), and the ASD-Integrity factors F(1, 470) = 4.475, p = .035). Men endorsed more positive attitudes than women on both the Acceptability and Integrity factors. Women endorsed more positive attitudes on the Instrumental factor. Similar ANOVAs were conducted across the three Fraboni Scale of Ageism factors. Significant differences between men and women were only obtained across the Avoidance factor (F(1, 470) = 12.187, p = .001) with endorsing higher avoidance scores than women. Taken together these results show differential effects across three ASD factors between men and women while men demonstrated higher scores on the FSA Avoidance scale.

